# Transforaminal Endoscopic Lumbar Foraminotomy for Radiculopathy at the Fused Segment After Lumbar Fusion: Clinical Outcomes and Surgical Considerations

**DOI:** 10.3390/jcm15124789

**Published:** 2026-06-20

**Authors:** Yong Ahn, Han-Byeol Park, Sung-Ho Do, Sojung Lee

**Affiliations:** 1 Department of Neurosurgery, Kyung Hee University Hospital at Gangdong, Kyung Hee University College of Medicine, Seoul 05278, Republic of Korea; onlytheone86@naver.com; 2 Department of Neurosurgery, Gachon University Gil Medical Center, Incheon 21565, Republic of Korea; phbsgood@gilhospital.com; 3 Independent Researcher, Atlanta, GA 30322, USA; sojunglee0618@gmail.com

**Keywords:** lumbar vertebrae, spinal fusion, radiculopathy, foraminal stenosis, endoscopy, minimally invasive surgical procedures

## Abstract

**Background:** Radiculopathy originating from a previously fused lumbar segment is a clinically relevant but often underrecognized problem. Progressive foraminal stenosis may develop due to postoperative structural changes, leading to mechanical irritation of the exiting nerve root. Transforaminal endoscopic lumbar foraminotomy (TELF) is a minimally invasive option, but its role in this setting is not well defined. **Methods:** In this retrospective cohort study, we included 36 consecutive patients who underwent TELF for symptomatic foraminal stenosis at a previously fused segment between 2020 and 2023. Clinical outcomes were assessed using the visual analog scale (VAS) for leg pain, Oswestry Disability Index (ODI), and modified MacNab criteria, with follow-up of up to 2 years. Radiographic and intraoperative findings were reviewed to explore the underlying mechanisms. **Results:** The mean VAS score improved significantly from 8.36 preoperatively to 2.00 at 2 years, and the mean ODI decreased from 70.9% to 16.8%. According to the modified MacNab criteria, 86.1% of the patients achieved excellent or good outcomes. Intraoperative findings revealed fibrotic or hypertrophic foraminal stenosis in 86.1% patients (n = 31), whereas 13.9% of patients (n = 5) showed pedicle screw-related nerve root irritation. Five patients experienced transient postoperative dysesthesia, and no postoperative instability was observed. **Conclusions:** Radiculopathy at the fused segment is primarily caused by progressive mechanical foraminal compromise after fusion. TELF provides effective symptom relief through direct decompression and may serve as a less invasive alternative to revision fusion in selected patients.

## 1. Introduction

Lumbar fusion is widely performed for treating degenerative lumbar spinal disorders. However, persistent or newly developed radiculopathy following fusion remains a challenging clinical problem [1,2,3]. Although adjacent segment disease has been extensively studied, radiculopathy originating from the fused level, particularly progressive foraminal stenosis, has received less attention [4,5,6]. In contrast to primary foraminal stenosis, post-fusion stenosis of the fused segment develops in a mechanically altered and instrumented environment. Postoperative structural changes, including segmental subsidence or incomplete fusion, may progressively narrow the foraminal space and lead to mechanical irritation of the exiting nerve root. Rather than representing discrete pathological subtypes, this condition is more consistently understood as a result of progressive mechanical compromise within the fused segment. Revision surgery in this setting often requires wide exposure, hardware removal, extensive decompression, and reinstrumentation procedures, which may increase surgical morbidity and complication risk [7,8,9,10,11,12,13].

Transforaminal endoscopic lumbar foraminotomy (TELF) has been established as an effective minimally invasive technique for lumbar foraminal stenosis, allowing for targeted neural decompression while preserving the posterior stabilizing structures [14,15,16,17,18]. Since the transforaminal approach avoids posterior scar tissue and implanted hardware, TELF might be a valuable option for post-fusion radiculopathy occurring at either the adjacent levels [19] or fused segment itself [20]. However, the clinical role of TELF in radiculopathy arising from the fused segment, particularly under mechanically altered conditions, remains insufficiently defined. Based on our clinical experience and literature reviews, radiculopathy at the fused segment is most commonly associated with progressive foraminal narrowing caused by incomplete fusion or postoperative degenerative changes [21,22,23,24,25,26]. From a surgical perspective, we applied an advanced form of TELF based on the concept of extended decompression. This technique emphasizes hardware-avoiding transforaminal access, pedicle-to-pedicle bony decompression, and circumferential release of the exiting nerve root within the foraminal corridor.

We aimed to evaluate the clinical outcomes of advanced TELF for foraminal stenosis at the fused segment after lumbar fusion and to describe the key mechanical factors and practical endoscopic strategies for effective neural decompression in this setting.

## 2. Materials and Methods

### 2.1. Patient Population and Eligibility Criteria

**Figure 1:**
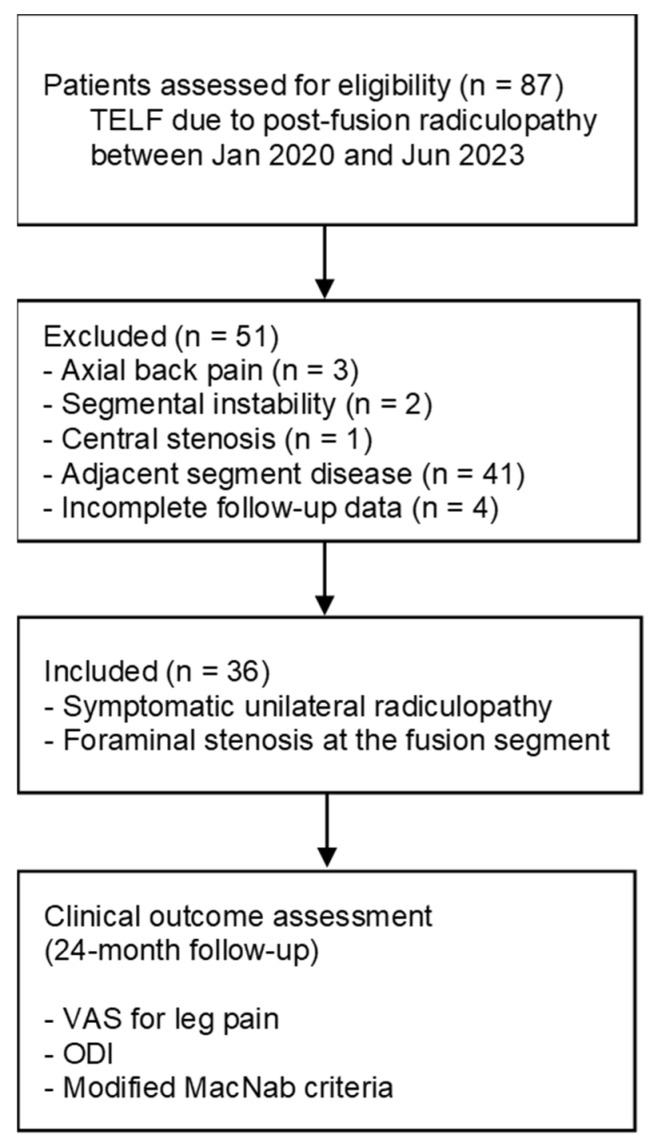
Flowchart of patient screening, exclusion, and final cohort selection for the study. TELF, transforaminal endoscopic lumbar foraminotomy; VAS, visual analog scale; ODI, Oswestry Disability Index.

This retrospective cohort study analyzed a prospectively maintained database of consecutive patients who underwent TELF for symptomatic foraminal stenosis at the fused segment after lumbar fusion surgery (same-segment pathology). Institutional Review Board approval was obtained before data extraction (GDIRB2023-210; 24 June 2023); the requirement for informed consent was waived owing to the retrospective nature of the analysis and use of de-identified data. Between January 2020 and June 2023, 36 patients underwent TELF for persistent unilateral radiculopathy attributable to foraminal pathology at a previously fused lumbar level. The inclusion criteria were: (1) severe radicular leg pain despite at least 6 months of structured non-operative management; (2) a pain-free interval of at least 6 months after the initial lumbar fusion; (3) exiting nerve root compression due to foraminal stenosis [27,28] at the fused segment confirmed by computed tomography (CT) and magnetic resonance imaging (MRI); (4) absence of significant segmental instability on dynamic radiographs, defined as translation of >3 mm or angular motion of >10° on flexion–extension views; (5) concordance between symptoms, dermatomal distribution, and neurological findings; and (6) symptom reproduction and/or temporary relief after selective nerve root block. Patients were excluded if they had predominant axial back pain, clear instability requiring revision fusion, central canal stenosis as the primary pathology, infection, tumors, or incomplete follow-up data. Three patients were excluded because of incomplete 24-month follow-up data. Baseline variables included age, sex, fusion level, time interval from fusion to TELF, and surgical characteristics. The patient selection process is summarized in Figure 1.

### 2.2. Surgical Techniques

All procedures were performed using a percutaneous transforaminal endoscopic approach under local anesthesia and conscious sedation. The patients were positioned prone on a radiolucent table with slight hip and knee flexion. Preoperative imaging findings were carefully reviewed to determine the optimal skin entry point and trajectory, particularly in relation to the existing instrumentation.

A fluoroscopy-guided outside-in approach was adopted for safely accessing the foraminal zone while minimizing irritation to the exiting nerve root. A relatively steep posterolateral trajectory was used to facilitate safe foraminal access and decompression. The initial needle was directed toward the inferior surface of the superior articular process or the posterior vertebral body, depending on the fused anatomy and hardware configuration. The working sheath was positioned in the outer foraminal zone without traversing the nerve root corridor.

Endoscopic decompression began with systematic bony unroofing of the foramen. The hypertrophic tip of the superior articular process and associated osteophytes were undercut using endoscopic burrs until the foraminal ligament and ligamentum flavum were adequately exposed. In cases of a rigid fusion anatomy, bony decompression was extended vertically along the pedicle walls to secure a sufficient resection margin around the exiting nerve root. Particular attention was paid to identify reliable bony landmarks, including the disc surface, pedicle borders, facet joint synovium, and isthmus, because orientation within the scarred fusion bed can be difficult. Following adequate bone resection, soft tissue decompression was performed under direct endoscopic visualization. Hypertrophic ligamentum flavum, foraminal ligaments, fibrotic adhesions, and disc or cystic fragments were removed using micropunches, forceps, and radiofrequency probes. Decompression was directed proximally toward the axillary epidural zone and distally toward the lateral exit zone along the anatomical course of the exiting nerve root, rather than parallel to the disc space. In cases of screw-related neural irritation, decompression was primarily focused on carefully dissecting and separating the exiting nerve root from the screw threads to achieve adequate neural release. The endpoint of decompression was defined as circumferential release and free mobilization of the exiting nerve root from the proximal axillary zone to the lateral exit zone within an adequately enlarged foramen.

**Figure 2:**
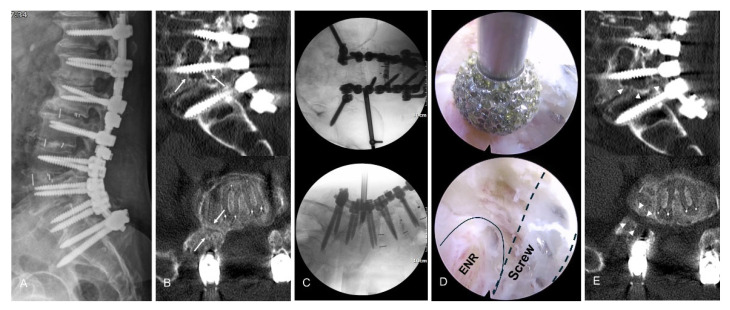
Illustrative case of fused segment lumbar foraminal stenosis. A 69-year-old man presented with right L5 radiculopathy after multiple prior lumbar decompression and fusion surgeries. (**A**) Plain radiograph showing multi-level lumbar fusion. (**B**) Preoperative CT demonstrating right L5–S1 foraminal stenosis and intrusion of the L5 pedicle screw into the foramen (arrows). A right L5 nerve root block provided only temporary relief. (**C**) TELF was performed, with the working sheath positioned in the right L5–S1 foraminal zone. (**D**) Endoscopic view showing exposed pedicle screw threads (dotted area) irritating the ENR. Endoscopic unroofing and extended foraminal decompression achieved full ENR release. (**E**) Postoperative CT showing adequate foraminal decompression (arrowheads). CT, computed tomography; ENR, exiting nerve root; TELF, transforaminal endoscopic lumbar foraminotomy.

Postoperative MRI or CT images were selectively performed when residual compression was suspected or when atypical postoperative symptoms occurred (Figure 2).

### 2.3. Clinical Outcome Measures

Clinical outcomes were assessed preoperatively and at 6 weeks, 6 months, 1 year, and 24 months postoperatively. All included patients completed the 24-month follow-up evaluation. Leg pain intensity was measured by the visual analog scale (VAS); functional disability was measured by the Oswestry Disability Index (ODI) [29]. Global clinical outcomes at the final follow-up were assessed by the modified MacNab criteria [30], which categorized outcomes as excellent (complete resolution of pain and unrestricted activity), good (occasional nonradicular pain with substantial relief of presenting symptoms), fair (some functional improvement but persistent limitations), or poor (insufficient improvement or need for further intervention). Excellent or good outcomes were defined as successful clinical results. Perioperative adverse events, including postoperative dysesthesia, were tracked; furthermore, the duration of hospitalization was recorded. The 6-week follow-up was selected as the first formal postoperative evaluation point to minimize the influence of immediate postoperative pain fluctuation and transient dysesthesia.

### 2.4. Statistical Analysis

Statistical analyses were performed using SPSS software (version 22.0; IBM Corp., Armonk, NY, USA). Continuous variables are presented as mean ± standard deviation, whereas categorical variables are presented as frequencies and percentages. Normality of continuous variables was assessed using the Shapiro–Wilk test. Changes in VAS and ODI scores over time were analyzed using repeated-measures analysis of variance with Bonferroni correction for multiple comparisons. Preoperative and final follow-up values were additionally compared using paired *t*-tests. Categorical variables were analyzed using Fisher’s exact test where appropriate. Mean changes and 95% confidence intervals (CIs) were calculated for the major clinical outcomes. A *p* value < 0.05 was considered statistically significant.

## 3. Results

### 3.1. Baseline Characteristics and Perioperative Course

A total of 36 patients with 24-month follow-up were included in the final analysis. The mean age was 65.8 ± 9.3 years (range, 43–84), and 55.6% of patients (n = 20) were women. The most frequently treated level was L4–5 (47.2%), followed by L5–S1 (36.1%) and L3–4 (16.7%). The mean interval between the index fusion and onset of symptomatic foraminal stenosis was 34.8 ± 25.7 months. All procedures were successfully completed using a transforaminal endoscopic approach under local anesthesia. The mean operative time was 59.7 ± 15.1 min, and the mean hospital stay was 1.53 ± 0.74 days. No patients required intraoperative conversion to open revision surgery. Detailed baseline characteristics and perioperative variables are summarized in Table 1.

### 3.2. Clinical Outcomes

**Figure 3:**
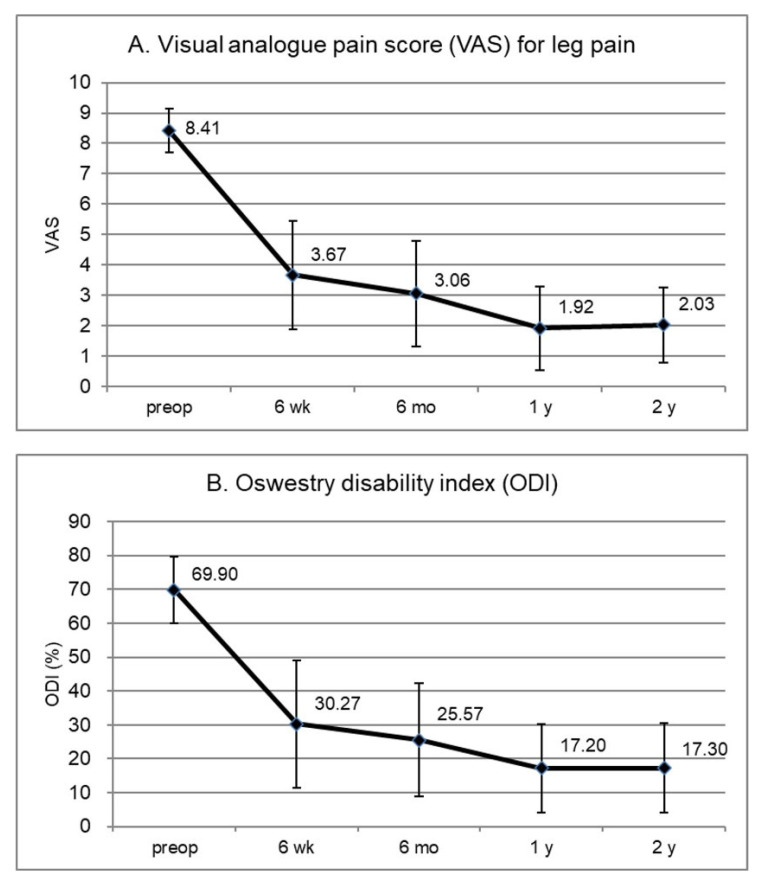
Clinical outcomes after TELF for fused-segment radiculopathy after lumbar fusion. (**A**) Leg pain measured by VAS significantly improved over time. (**B**) Functional disability measured by ODI also showed marked improvement during follow-up. TELF, transforaminal endoscopic lumbar foraminotomy; VAS, visual analog scale; ODI, Oswestry Disability Index.

Radicular pain improved after surgery. The mean leg VAS decreased from 8.36 ± 0.72 preoperatively to 3.67 ± 1.79 at 6 weeks and further improved to 2.00 ± 1.12 at the 2-year follow-up (Figure 3A). Functional disability also improved, with the mean ODI decreasing from 70.86 ± 10.13% preoperatively to 16.84 ± 12.59% at 2 years postoperatively (Figure 3B).

**Figure 4:**
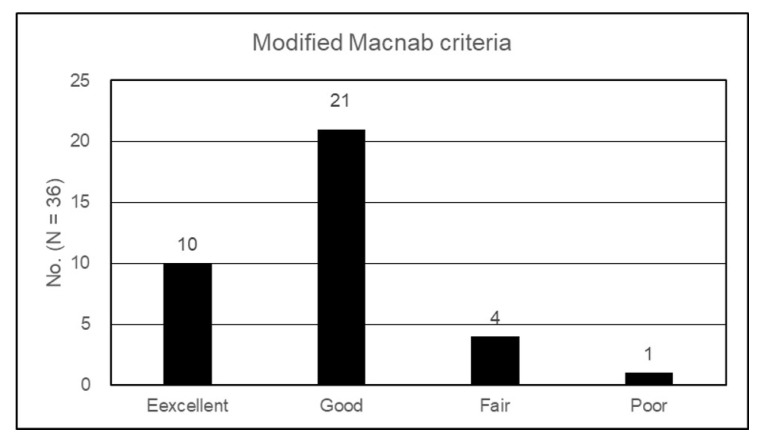
Global clinical outcomes according to the modified MacNab criteria. Excellent or good outcomes were achieved in 86.11% of the patients at the final follow-up.

According to the modified MacNab criteria, 27.8% of patients (n = 10) achieved excellent outcomes and 58.3% (n = 21) achieved good outcomes, resulting in an overall clinical success rate of 86.1% (95% CI, 71.3–93.9%) (Figure 4). Four patients (11.1%) were classified as fair and one (2.8%) as poor. One patient with persistent radiculopathy after L3–4 TELF subsequently underwent revision fusion surgery at another institution and was classified as having a poor outcome.

Postoperative complications were infrequent and minor. Transient postoperative dysesthesia occurred in 13.9% of patients (n = 5) and resolved with conservative management within 8–12 weeks. No major neurological deterioration, infection, dural tear, or implant-related failure were observed. Radiographic follow-up using dynamic flexion–extension views revealed preserved segmental stability. None of the patients developed new instability or progressive spondylolisthesis during follow-up.

### 3.3. Intraoperative and Radiologic Findings

**Figure 5:**
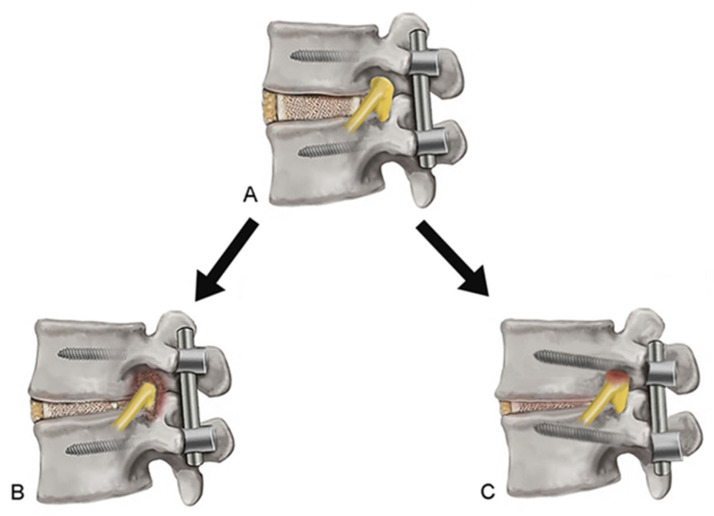
Pathophysiological mechanisms of fused-segment foraminal stenosis after lumbar fusion. (**A**) Immediate postoperative configuration after fusion. Over time, disc space subsidence and micromotion related to fusion failure may develop, leading to pathological changes, as shown in (**B**,**C**). (**B**) Foraminal stenosis caused by postoperative adhesions and facet hypertrophy. (**C**) Nerve root irritation caused by pedicle screw prominence associated with disc space subsidence.

Intraoperative findings revealed fibrotic or hypertrophic foraminal stenosis in 86.1% of patients (n = 31), whereas 13.9% of patients (n = 5) showed pedicle screw-related nerve root irritation. These findings were frequently associated with postoperative structural changes, such as segmental subsidence or incomplete fusion, leading to progressive stenosis and mechanical irritation of the exiting nerve root (Figure 5).

An exploratory subgroup analysis based on intraoperative phenotype is summarized in Table 2. Patients with screw-related irritation showed a 100% success rate (5/5), whereas those with fibrotic adhesion demonstrated an 83.9% success rate (26/31); however, this difference was not statistically significant (*p* = 0.55). Postoperative dysesthesia occurred in 12.9% of the fibrotic group and 20.0% of the hardware-related group, without a significant difference (*p* = 1.00).

## 4. Discussion

### 4.1. Main Findings and Clinical Implications

Radiculopathy arising at the fused segment after lumbar fusion is an underrecognized but clinically challenging condition. In the present study, TELF resulted in significant and durable symptom relief for this pathology. Radicular pain improved markedly, with the mean leg VAS decreasing from 8.36 preoperatively to 2.00 at the 2-year follow-up, while functional disability improved from 70.9% to 16.8%. According to the modified MacNab criteria, 86.1% of the patients achieved excellent or good outcomes. These findings indicated that mechanism-oriented endoscopic decompression could be an effective treatment option for managing carefully selected patients with post-fusion foraminal stenosis. Our results demonstrated that clinical improvement was highly significant across all primary metrics, including pain intensity, functional disability, and global clinical status. The mean VAS score for radicular pain improved by 6.33 (75.7%); the mean ODI improved by 45.57 (75.2%) at the final follow-up (*p* < 0.001). According to published articles, a reduction of >50% in VAS scores [31] and >30% in ODI [32,33] are generally considered clinically notable improvements. The improvement found in our cohort clearly exceeded the commonly accepted minimal clinically important difference thresholds, supporting the clinical efficacy of TELF for complex post-fusion foraminal pathology.

Traditionally, radiculopathy after lumbar fusion has been treated with open revision surgery. However, revision procedures frequently require extensive posterior exposure through the scar tissue, and might involve hardware removal or extension of the fusion construct, which could increase surgical morbidity [7,8,9,10,11,12,13]. In contrast, the transforaminal endoscopic approach allows direct access to the foraminal pathology while bypassing the posterior scar tissue and existing instrumentation. Our results demonstrated that targeted endoscopic decompression could achieve substantial clinical improvement while preserving the previous fusion construct. The consistent reduction in pain and disability scores (Figure 3) and high proportion of favorable global outcomes (Figure 4) suggest that TELF represents a useful minimally invasive treatment option for carefully selected patients. However, because this study did not include a direct comparison group, the present findings should not be interpreted as evidence of superiority over conventional revision surgery.

### 4.2. Reinterpretation of Pathophysiology: A Mechanical Perspective

Although radiculopathy at the fused segment has often been described as a heterogeneous condition, our observations suggest that a unified mechanism may better explain most of the cases (Figure 5). Following lumbar fusion, the operated segment exists in a structurally altered environment. In cases of incomplete or failed fusion, segmental micromotion, subsidence, and progressive peri-foraminal remodeling may develop over time [4,5,6]. In many cases, the pathology appeared to reflect localized mechanical failure within a relatively stable fused segment, rather than gross instability requiring formal revision fusion. These changes can gradually reduce foraminal height and width, leading to mechanical compression or irritation of the exiting nerve root.

From a clinical perspective, this condition is more consistently explained as a continuum of mechanical compromise, rather than distinct pathological subtypes. This concept provides a simpler and more practical framework for both diagnosis and surgical planning. Although subgroup-based interpretation was not the primary focus of this study, exploratory analysis showed a trend toward favorable outcomes in cases of pedicle screw-related irritation (Table 2). However, given the small sample size and lack of statistical significance, these findings should be interpreted with caution and do not contradict the overall mechanical concept.

### 4.3. Rationale and Advantages of TELF

Surgical management traditionally involves open revision procedures when radiculopathy develops in a fused lumbar segment. Although effective, revision surgery often requires extensive posterior exposure through the scar tissue and may involve hardware removal or extension of the fusion construct. These procedures can increase surgical morbidity and recovery time in selected patients [7,8,9,10,11,12,13]. In contrast, the transforaminal endoscopic approach provides direct access to the foraminal pathology while avoiding posterior scar tissue and existing instrumentation [34,35]. This approach allowed for the targeted decompression of the exiting nerve root while preserving the previous fusion construct. Although hardware removal may be necessary in severe cases of screw-related irritation, most patients in the present series achieved adequate symptom relief through endoscopic decompression and neural release without revision instrumentation. Another advantage of TELF is the ability to achieve direct neural decompression under endoscopic visualization. In the post-fusion foramen, neural compression is often multifactorial and may involve bony narrowing, fibrotic adhesions, and focal mechanical irritation. Therefore, effective decompression requires not only removal of focal compressive lesions but also circumferential release of the exiting nerve root within the foraminal corridor. Because TELF allows for simultaneous diagnostic exploration and targeted decompression through a minimally invasive transforaminal corridor, it may be a useful treatment option for selected patients with foraminal-dominant radiculopathy after lumbar fusion. However, because this study did not include a direct comparison group, the present findings should be interpreted cautiously and should not be considered evidence of superiority over conventional revision surgery.

### 4.4. Technical Tips: Landmark-Based Transforaminal Decompression for Circumferential Nerve Root Release

Although traditional TELF is recognized as an effective surgical method for treating lumbar foraminal stenosis, it still poses challenges for novice endoscopic surgeons attempting to replicate it in practical settings. With respect to many technical descriptions, the key issue of endoscopic orientation has not been sufficiently emphasized. Since the post-fusion foramen is narrow, scarred, and anatomically distorted, loss of orientation can easily lead to incomplete decompression or unnecessary neural irritation.

**Figure 6:**
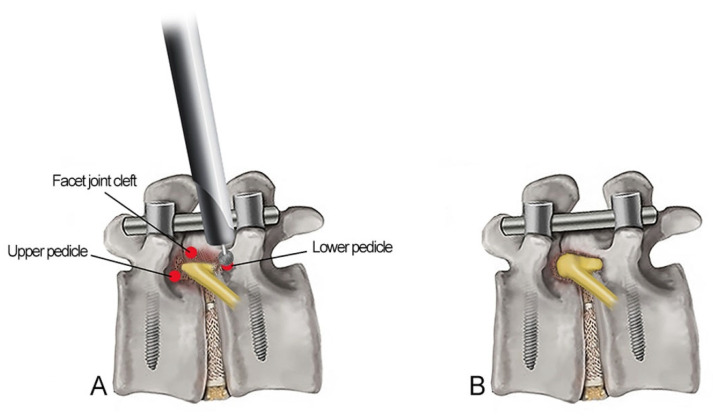
Landmark-based decompression strategy for advanced TELF. (**A**) Stepwise decompression guided by fixed bony landmarks (red dots indicating key anatomical landmarks). Decompression begins at the lower pedicle, proceeds along the superior articular process to identify the facet joint cleft, and continues toward the upper pedicle, achieving circumferential decompression of the ENR. (**B**) Endoscopic view after the final decompression showing a freely mobilized ENR within the enlarged foramen. TELF, transforaminal endoscopic lumbar foraminotomy; ENR, exiting nerve root.

The advanced or extended TELF technique used in this study was designed to make decompression more reproducible by maintaining a constant endoscopic orientation throughout the procedure and achieving circumferential decompression with sufficient resection margins [36,37,38]. Rather than relying primarily on soft tissue or subjective visual impressions, this method used clear bone landmarks. In our experience, decompression based on fixed osseous margins is easier to understand, more consistent and more reliable than a less-structured approach (Figure 6).

The basic concept is circumferential neural release with sufficient decompression margins (“from pedicle to pedicle,” “from axillary to shoulder,” and “from proximal to distal”). The procedure usually begins at the lower pedicle margin, which provides a safe and stable starting point for orientation. Decompression was performed along the superior articular process. As drilling continues, surgeons must identify the facet joint cleft, which is an important landmark. Further drilling through this area leads to the axillary zone of the exiting nerve root where proximal compression is often encountered. Undercutting the isthmus and upper pedicle allows for the decompression of the shoulder portion of the exiting nerve root. After adequate decompression of the shoulder-axillary portion, attention was directed to the more distal part of the foramen. Surgeons can achieve circumferential release of the nerve root, rather than simple focal exposure, by sequentially enlarging the foraminal corridor from the lower pedicle margin to the upper pedicle. This stepwise landmark-based strategy makes the advanced TELF technique more practical for post-fusion spines. By maintaining orientation and following a reproducible bone roadmap, the surgeon can perform a 360° circumferential decompression of the exiting nerve root in a safer and more consistent manner (Figure 6).

### 4.5. Future Perspectives

Despite the promising outcomes of extended TELF, specific technical barriers must be addressed to further optimize the procedure. Three primary challenges—the surgical approach, optics, and surgical instruments—remain critical areas for future improvement. Among these, advancements in surgical instruments are the most urgent. The development of steerable and articulating endoscopic instruments would significantly enhance the surgeon’s ability to navigate within the confined transforaminal space, allowing for more effective decompression with a reduced risk of neural injury [38]. In addition, innovations in optical systems, including high-resolution endoscopic imaging and real-time three-dimensional visualization, may further improve the intraoperative precision. As technology evolves, the integration of robotic assistance and navigation systems driven by artificial intelligence holds promise for overcoming the current limitations and reducing the learning curve associated with endoscopic spine surgery.

### 4.6. Study Limitations

This study has some limitations. First, it was a retrospective study conducted at a single institution with a relatively small sample size, which may limit the generalizability of the findings. Because radiculopathy at the fused segment is a relatively uncommon and selective condition, only patients with complete 24-month follow-up were included in the final analysis. Second, the absence of a control group precluded a direct comparison with conventional revision surgery. Therefore, the present findings should be interpreted with caution. Third, no formal sample size calculation was performed because of the exploratory retrospective design. In addition, detailed subgroup analyses were limited by the small number of patients in each subgroup. Detailed systemic comorbidity and body mass index data were not consistently available in this retrospective cohort and therefore were not included in the analysis. Future studies with larger cohorts and comparative designs are needed to further validate these findings and refine surgical indications.

## 5. Conclusions

Radiculopathy at the fused segment is primarily a mechanical problem caused by progressive foraminal compromise after fusion. In this study, TELF provided notable improvement in radicular pain and functional disability with low morbidity in carefully selected patients. The transforaminal endoscopic approach allowed for direct decompression of the exiting nerve root while preserving the existing fusion construct and avoiding extensive revision surgery. These findings suggest that TELF may be a useful minimally invasive option for treating fused-segment foraminal radiculopathy under mechanically altered post-fusion conditions. Further comparative studies with larger cohorts are needed to validate these findings and refine surgical indications.

## Figures and Tables

**Table 1:** Baseline characteristics and perioperative data.

Variable (n = 36)		Value
Age (yr)		65.8 ± 9.3 (range 43–84)
Sex	female	20 (55.6%)
	male	16 (44.4%)
Treated level	L3–4	6 (16.7%)
	L4–5	17 (47.2%)
	L5–S1	13 (36.1%)
Fusion-to-TELF interval		34.8 ± 25.7 months (0.33–7.0 years)
Previous fusion	Single level	27 (75%)
	Multi-level	9 (25.0%)
Operative time (min)		59.7 ± 15.1 (range 35–96)
Hospital stay (days)		1.53 ± 0.74 (range 1–3)
Baseline leg pain (VAS)		8.36 ± 0.72 (range 7–10)
Baseline disability (ODI, %)		69.90 ± 6.84

TELF, transforaminal endoscopic lumbar foraminotomy; VAS, visual analog scale; ODI, Oswestry Disability Index.

**Table 2:** Clinical outcomes and complications according to pathophysiological phenotype.

Pathophysiological Phenotype	Total (n = 36)	Success	Failure	Dysesthesia
Fibrotic adhesion	31 (86.1%)	26 (83.9%)	5	4
Screw-related	5 (13.9%)	5 (100%)	0	1
Total	36 (100%)	31 (86.1%)	5	5
*p*-value		0.55		1.00

Success was defined as an excellent or good outcome according to the modified MacNab criteria.

## Data Availability

The data presented in this study are available upon request from the corresponding author.

## Abbreviations

The following abbreviations are used in this manuscript:

CT	Computed tomography
ENR	Exiting nerve root
MRI	Magnetic resonance imaging
ODI	Oswestry Disability Index
TELF	Transforaminal Endoscopic Lumbar Foraminotomy
VAS	Visual Analog Scale
